# Narrow-Margin Excision for Invasive Acral Melanoma: Is It Acceptable?

**DOI:** 10.3390/jcm9072266

**Published:** 2020-07-16

**Authors:** Takamichi Ito, Yumiko Kaku-Ito, Maiko Wada-Ohno, Masutaka Furue

**Affiliations:** Department of Dermatology, Graduate School of Medical Sciences, Kyushu University, 3-1-1, Maidashi, Higashi-ku, Fukuoka 812-8582, Japan; kyumiko@dermatol.med.kyushu-u.ac.jp (Y.K.-I.); maiko@dermatol.med.kyushu-u.ac.jp (M.W.-O.); furue@dermatol.med.kyushu-u.ac.jp (M.F.)

**Keywords:** surgery, acral lentiginous melanoma, prognosis

## Abstract

In this retrospective review of 100 patients with primary invasive acral melanoma, we examined whether narrow-margin excision is warranted for acral melanoma. Patients treated with surgical margins recommended by the National Comprehensive Cancer Network (R-group) were compared to those treated with narrow margins (N-group). A total of 65 patients underwent narrow-margin excision. Positive margin status or local recurrence rarely occurred regardless of the excision margins, whereas fatal events frequently occurred, particularly among the patients with T4 melanoma. The mortality rates of N- and R-group with T1–3 melanomas were similar (1.36 and 1.28 per 100 person-years, respectively). However, patients with T4 melanoma treated with narrow-margin excision had a higher mortality rate (11.44 vs. 5.03 per 100 person-years). Kaplan–Meier analyses showed a worse prognosis in the N-group (*p* = 0.045) but this group had thicker Breslow thickness (4.21 mm vs. 2.03 mm, *p* = 0.0013). A multivariate analysis showed that Breslow thickness was an independent risk factor, but surgical margin was not a risk factor for melanoma-specific survival or disease-free survival. In conclusion, although we could not find a difference between the narrow-margin excision and recommended-margin excision in this study, we suggest following current recommendations of guidelines. Our study warrants the prospective collection of data on acral melanoma to better define the prognosis of this infrequent type of melanoma.

## 1. Introduction

Malignant melanoma is an aggressive tumor that mostly arises on the skin. The incidence of this disease is increasing worldwide [1,2]. Clinical application of targeted therapy (BRAF inhibitors with or without MEK inhibitors) and immunotherapy (PD-1 inhibitors and CTLA-4 inhibitors) improve the survival of patients with unresectable melanoma, but the principal therapy for localized melanoma is surgical eradication [3,4]. To achieve complete excision of primary melanoma, wide-margin excision (e.g., 5 cm around the tumor) has historically been performed [5,6,7]. However, extensive excision inevitably involves unnecessary removal of normal tissue, causing large skin defects. In such cases, skin grafting or skin flaps, rather than simple suturing, may be needed to close the defects. Many randomized controlled studies have been conducted to explore appropriate surgical margins for melanoma [5,8,9,10,11,12]. A large randomized controlled study (*n* = 936) found no significant differences in either overall survival or recurrence-free survival between patients with thick melanoma (>4 mm) who underwent resections with 2-cm or 4-cm margins [12]. Another large randomized controlled study compared the overall survival between patients undergoing 1-cm and 3-cm resections. Although the 1-cm excision group had a higher risk of locoregional recurrence, overall survival was similar to the 3-cm excision group [11]. The latest National Comprehensive Cancer Network (NCCN) guidelines recommend surgical margins according to Breslow thickness of the primary melanoma: 5 mm for in situ melanoma, 10 mm for T1 melanoma, 10–20 mm for T2 melanoma, and 20 mm for T3 and T4 melanoma [3].

**Figure 1:**
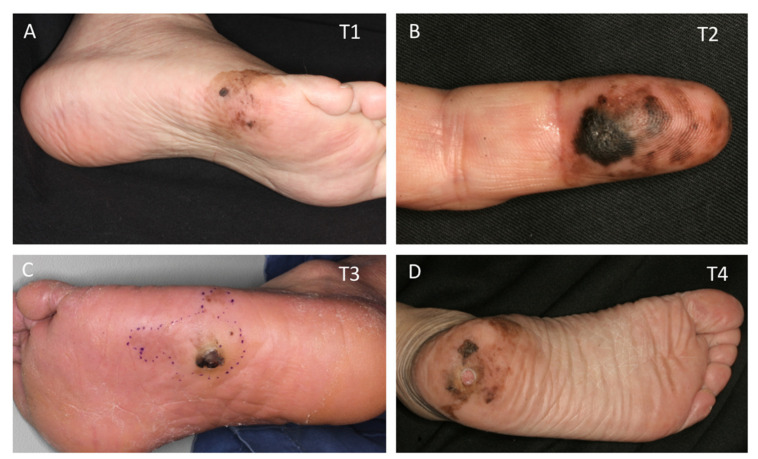
Acral melanoma: T1 (**A**), T2 (**B**), T3 (**C**), and T4 (**D**). All show a characteristic lentiginous spread of melanoma cells (acral lentiginous melanoma) with brown, irregularly shaped, wide-spreading macules.

Acral melanoma is a type of cutaneous melanoma that occurs on the glabrous skin of the palms, soles, and nail beds. It has a unique genetic background compared to other types of melanoma [13,14]. Histopathologically, most acral melanomas are of the acral lentiginous subtype, characterized by the lentiginous spread of melanoma cells in the basal layer of the epidermis [15,16]. Acral melanoma may show a relatively small nodule surrounded by a wide spreading in-situ macule (Figure 1).

**Figure 2:**
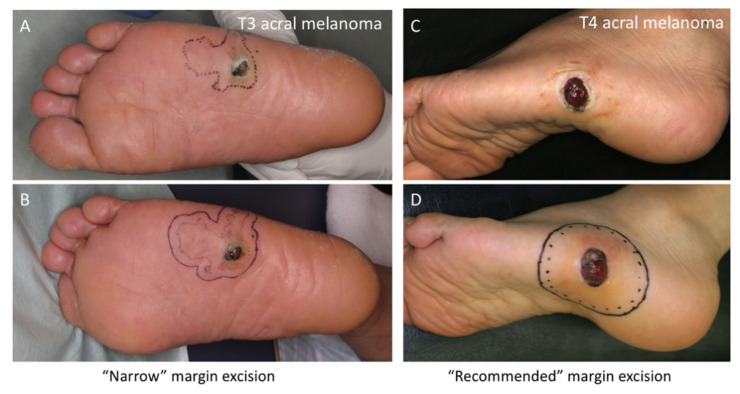
T3 acral melanoma with narrow excision margins on the sole (**A** and **B**). The dashed lines indicate the clinical tumor border. The solid resection line at the time of excision biopsy was set 0.5 cm away from the dashed line, and a final surgical margin of 1 cm was achieved at the time of reconstruction. Although the final margin was 1 cm away from the macule, it was 2 cm from the nodule. T4 acral melanoma with recommended excision margins (**C** and **D**). The dashed line indicates a 2-cm margin, and the solid resection line is set 2.5 cm away from the nodule.

Sufficient surgical margins for acral melanomas may not always be feasible due to anatomical constraints. Extensive excision for melanoma in the nail beds causes amputation at more proximal sites. For melanoma of the soles or palms, reconstruction requires wider skin grafting in weight-bearing areas, leading to long-lasting pain. When designing the surgical resection line for acral melanoma, the question is: How far away from the surrounding (seemingly) in situ macule should the resection line be? Theoretically, melanoma cells in situ are restricted within the epidermis and do not metastasize. Figure 2A shows a typical case of T3 acral melanoma with a wide spreading in situ area. Although the ideal resection line is 2 cm away from the in-situ area, such wide excision could be an overtreatment. In this case, we excised with a final margin of 1 cm around the surrounding macule (0.5 cm at the time of excision biopsy, as shown in Figure 2B, and an additional 0.5 cm at the time of reconstruction). Although the final margin was 1 cm away from the macule, it was 2 cm away from the nodule. This resection appears to be appropriate, and we have sometimes chosen this kind of narrow-margin excision for acral melanoma after in-depth discussion with patients. However, another question arises: Are narrow-margin excisions warranted? For comparison, Figure 2C,D shows a case in which the acral melanoma was excised with recommended surgical margins from the macule. In this article, we summarize our 18 years of experience treating acral melanomas and analyze the outcomes of narrow-margin excisions. One hundred patients with invasive acral melanoma and long-term follow-up are included.

## 2. Materials and Methods

### 2.1. Ethics Statement

We conducted this retrospective study in accordance with the concepts enshrined in the Declaration of Helsinki. This study was approved by the Kyushu University Institutional Ethics Committee (30-363; 27 November 2018).

### 2.2. Patients

We identified 100 patients with primary invasive acral melanoma treated at Kyushu University Hospital’s Department of Dermatology between July 2001 and August 2018. Patients with in-situ acral melanoma were excluded. For all patients, at least three experienced dermatopathologists confirmed the diagnosis. Clinical and demographic data were retrieved from the patients’ files. Patients were treated and received follow-up in accordance with NCCN guidelines [3], except for the surgical margins of primary tumors. Sentinel lymph node biopsy and the subsequent completion lymph node dissection were performed for eligible patients. Preoperative CT with or without PET-CT were routinely performed for tumor staging.

Melanoma-specific survival (MSS) and disease-free survival (DFS) were calculated from the date of the first histopathological examination to the date of death as a result of melanoma and the date of any recurrence, respectively. Satellite and in-transit metastases were not regarded as local recurrences since they belong to category N. Data on patients without death or recurrence were censored on the date of the last follow-up, and data on patients who died of other causes were censored at the time of death.

### 2.3. Surgical Margins

To examine the impact of narrow-margin excisions on patient outcomes, we retrieved data regarding surgical margins at the initial surgery from patients’ files and operation records. Surgical margins were calculated from the lateral clinical borders including in situ macules. For lesions with a nodule surrounded by a wide macule, surgical margins from nodules were also recorded. For patients underwent excisional biopsy, the final surgical margins after the following wide local excision and reconstruction were recorded. For example, an excisional biopsy with a 3 mm margin followed by additional excision with a 7 mm margin was recorded as 10 mm of the final margin. Dermoscopy was used to improve the accuracy of tumor border detection after its clinical application. For single acral melanomas that were excised with inconsistent margins (e.g., 2 cm for the proximal side and 1 cm for the distal side), we recorded the minimum surgical margin from the tumor. Deep surgical margins were typically deeper layer of subcutaneous adipose tissue unless patients underwent amputation. Negative deep surgical margins were histopathologically confirmed in all lesions.

The Breslow thickness was histopathologically measured in all lesions. Patients were then divided into two groups according to the final surgical margins and the Breslow thickness. Patients underwent surgical excision with the final margins (measured from the lateral clinical borders including in situ macules) narrower than those recommended by the NCCN [3] were into the narrow-margin group (N-group hereafter), and patients with the final margins equal to (or wider than) the recommendation margins were into the recommended-margin group (R-group hereafter).

### 2.4. Statistical Analysis

All statistical analyses were performed using the JMP Pro statistical software package (version 14.0; SAS, Cary, NC, USA) and the GraphPad Prism statistical software package (version 6; GraphPad Software, San Diego, CA, USA), as reported previously [17,18,19]. To evaluate the association between two variables, χ^2^, Fisher’s exact or Mann–Whitney’s U tests were used as appropriate. The Kaplan–Meier method and the log-rank test were used to evaluate MSS and DFS. Cox multivariate analyses were used to assess the influence of the surgical margins on survival. A *p*-value less than 0.05 was considered to indicate statistical significance.

## 3. Results

### 3.1. Patient Data

Clinicopathological data of all patients with invasive acral melanoma are shown in Table 1. All patients were Japanese; 42 (42.0%) were male, and 58 (58.0%) were female. The mean age was 67.0 years (range: 16–89). All melanomas were histopathologically acral lentiginous melanomas with the epidermal component extending more than three rate ridges lateral to the dermal component. The sole was the predominant primary tumor site (65.0%), followed by the nail bed (20.0%) and the palm (15.0%). Ulceration was seen in 49 (49.0%) cases. There were 31.0%, 15.0%, 16.0%, and 38.0% of T1, T2, T3, and T4 acral melanomas, respectively. Seventy-two patients had localized disease (American Joint Committee on Cancer (AJCC) stage I or II) [20] and 28 had metastasized disease (AJCC stage III or IV).

### 3.2. Surgical Margins for Invasive Acral Melanomas

Table 2 summarizes the margin status of patients at the time of initial surgery. Many patients underwent surgical excision with margins narrower than those recommended by the NCCN [3] when the surgical margins were calculated from the lateral tumor borders including the surrounding in-situ macules. Interestingly, tumor-positive surgical margins were only noted in three patients: two in the R-group and one in the N-group. One patient with T1 melanoma resected with 1 cm margins underwent re-excision with an additional 5 mm margin and survived without any recurrence for 118 months. The other two patients did not want re-excision due to old age and were therefore observed without additional treatment. These patients did not demonstrate any recurrence during the follow-up period of 61 months and 19 months, respectively.

### 3.3. Patient Outcomes at the End of the Follow-Up Periods

We analyzed patient outcomes after follow-up, focusing on comparisons between the N- and R-groups (Table 3). Five patients with stage IV melanoma were excluded in this analysis because wide local excision for these patients may not be required and surgical margins are unlikely to influence on the survival; the remaining 95 patients were analyzed. Surprisingly, only two patients (2.1%) experienced local recurrence. Both were in the N-group (one with T1 melanoma and the other with T4) with a total follow-up of 269.6 person-years; none were in the R-group, with a total follow-up of 175.9 person-years. 

In contrast, 19 patients (20.0%) died of melanoma during the follow-up period. Of note, patients with T1–3 acral melanoma were less likely to die of melanoma regardless of the resection margin (four in 61, 6.6%), but death due to melanoma was concentrated in T4 acral melanoma patients, with nearly half of them dying (15 out of 34, 44.1%). For T1–3 acral melanoma patients, two of 31 patients in the N-group died during a total follow-up of 147.2 person-years, and two of 30 patients in the R-group died during a total follow-up of 156.0 person-years. There seems to be no major difference between the two groups in terms of the survival of T1–3 melanoma patients. For T4 acral melanoma patients, the results of the N-group were much worse (14 of 29 patients died) than those of the R-group (one of five patients died), granted the size of the latter group was small. Mortality rates or T4 N-group and T4 R-group were therefore 11.44 and 5.03 per 100 person-years, respectively.

### 3.4. Kaplan-Meier Analysis for MSS and DFS

**Figure 3:**
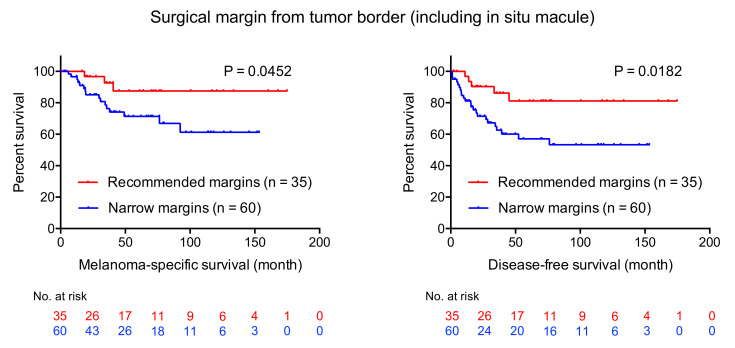
Kaplan-Meier survival curves for melanoma-specific survival (MSS) and disease-free survival (DFS). Narrow-margin excision is a significant factor for worse prognosis both for MSS and DFS when surgical margins were measured from the lateral tumor borders (5-year survival, 71.3% vs. 87.5%, 57.1% vs. 81.1%, *p* = 0.0452 and *p* = 0.0182, respectively).

To investigate the impact of narrow-margin excision on patient survival, Kaplan-Meier analyses were performed. Patients with stage IV melanoma were excluded from these survival analyses. Narrow-margin excision was a significant factor for worse prognosis both for MSS and DFS when surgical margins were measured from the lateral tumor borders (five-year survival, 71.3% vs. 87.5%, 57.1% vs. 81.1%, *p* = 0.0452 and *p* = 0.0182, respectively; see Figure 3). 

### 3.5. Comparison between Narrow and Recommended Margins

We next compared the background clinicopathological data between the N- and R-groups (Table 4). The former group had significantly thicker acral melanomas (*p* = 0.0013), while other factors, including age, sex, primary tumor site, presence of ulceration, and AJCC stages, were not significantly different between the two groups.

### 3.6. Prognostic Impact of Surgical Margin from Nodule

**Figure 4:**
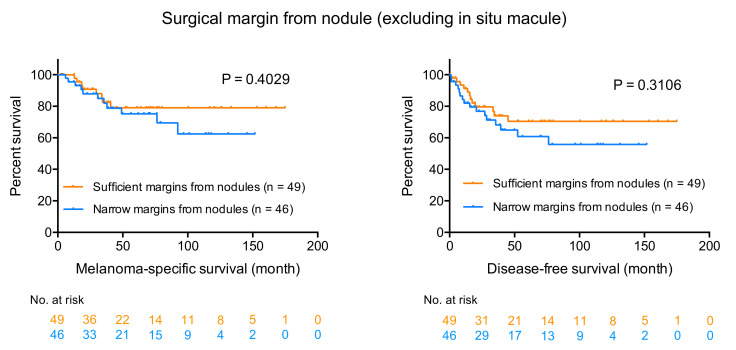
Kaplan-Meier survival curves of the “sufficient-margins from nodules” group and the “narrow-margins from nodules” group. Although the survival curves of the narrow margin group are under those of sufficient margin group, there is no statistical difference between the groups with sufficient and narrow margins (*p* = 0.4029 for MSS, and *p* = 0.3106 for DFS).

We then analyzed the prognostic impact of surgical margins from nodule or invasive area (excluding in situ macule). Patients with stage IV melanoma were excluded from these survival analyses. For all patients in the R-group, surgical margins from nodules or invasive areas were also sufficient (recommended-margins or more). However, among patients in the N-group, we found 14 patients (eight males and sic females; 3 T1, 2 T3, and 9 T4 melanomas) whose acral melanomas were resected with sufficient margins from nodules (Figure 2A,B shows an example of such cases). Seven patients experienced lymph node or distant metastasis, and 5 patients died of melanoma during the follow-up period. Figure 4 shows the Kaplan–Meier survival curves for MSS and DFS. Interestingly, there is no statistical difference between the groups with sufficient and narrow margins, although the survival curves of the narrow margin group are under those of the sufficient margin group (*p* = 0.4029 for MSS, and *p* = 0.3106 for DFS; see Figure 4).

### 3.7. Comparison between Narrow and Sufficient Margins from Nodules

We then checked the background clinicopathological data between the “sufficient-margin from nodule” group and the “narrow-margin from nodule” group (Table 5). There was no statistical difference between the two groups among the factors examined, although the narrow group tended to have thicker melanomas (*p* = 0.0633).

### 3.8. Cox Multivariate Analyses for MSS and DFS: Surgical Margins from Lateral Borders

We then assessed the prognostic impact of surgical margin (from lateral border) on MSS and DFS using Cox multivariate analyses (Table 6). The model covered the surgical margin as well as three variables (sex, age, Breslow thickness) since Breslow thickness was significantly different between the two groups. The surgical margin was not an independent prognostic factor either for MSS or DFS (Hazard ratio 1.83, 95% confidence interval 0.47–7.14, *p* = 0.3836; Hazard ratio 1.73, 95% confidence interval 0.60–4.99, *p* = 0.3092; respectively).

### 3.9. Cox Multivariate Analyses for MSS and DFS: Surgical Margins from Nodules

Lastly, we checked the prognostic impact of surgical margins from nodules on MSS and DFS (Table 7). Similarly, the model covered the surgical margin from nodules as well as three variables (sex, age, Breslow thickness). The margin from nodule was not an independent prognostic factor either for MSS or DFS (hazard ratio 1.29, 95% confidence interval 0.50–3.33, *p* = 0.5962; hazard ratio 1.23, 95% confidence interval 0.56–2.70, *p* = 0.6087; respectively).

## 4. Discussion

This retrospective study aimed to investigate whether the acral melanoma excisions we performed using narrow resection margins yielded results comparable to those we performed using recommended resection margins in terms of local recurrence and patient survival. Our findings were unexpected.

We observed considerably good primary disease control. Only three of 100 patients (3.0%) had tumor-positive margins regardless of resection with narrow (one patient) or recommended (two patients) margins. One patient underwent re-excision, but the other two did not due to advanced age; none experienced any local recurrence or distant metastasis. During the follow-up period, local recurrence occurred in only two patients (2.0%), who were both in the N-group. These results imply no remarkable impact on primary disease control between narrow and recommended margin excision.

In contrast to the good primary disease control, 19 patients (20.0%) died of melanoma among the 95 patients with stage I–III diseases. In the Kaplan–Meier survival curves for these patients, N-group patients had a significantly shorter MSS and DFS than R-group patients. This result should be interpreted with caution, however, because patients in the N-group had significantly thicker acral melanomas. Most death events occurred among T4 acral melanoma patients (78.9% of melanoma deaths, 15/19), and patients with T1–3 melanoma had relatively good prognoses. For patients with T1–3 melanoma, the mortality rate of the N-group was 1.36 per 100 person-years, and that of the R-group was 1.28 per 100 person-years, showing similar rates in the two group. For patients with T4 melanoma, the mortality rate of the N-group was 11.44 per 100 person-years, and that of the R-group was 5.03 per 100 person-years, suggesting a higher risk in the N-group. Interestingly, when adjusted by Breslow thickness in multivariate analyses, narrow-margin excision was not a significant prognostic factor either for MSS or DFS (*p* = 0.3836 and *p* = 0.3092, respectively). 

The results of the analyses on the surgical margins from nodules are also interesting and unexpected. Amazingly, the narrow-margin excision from nodules did not statistically impair MSS or DFS compared to the sufficient-margin excision from nodules. Multivariate analyses also revealed no statistical significance between the narrow and sufficient excision from nodules on MSS or DFS (*p* = 0.5962 and *p* = 0.6087, respectively).

These findings may suggest that treatments to control primary disease could be considered separately from those to prevent distant metastases and subsequent fatal events. In the current study, positive surgical margins were observed only in three patients and local recurrence only in two patients. Interpretation of the results on the survival is, however, more complicated and challenging. For thinner acral melanomas (T1–3), the mortality rates of the N- and R-groups were similar. The possibility still remains that narrow-margin excision does not affect patient survival, but there is no evidence at this time that narrow-margin excision is safe. For T4 acral melanoma, prognosis of the patients is generally worse. The fact that sufficient surgical margins from nodules did not improve patient survival might imply the limitation of the surgical treatment alone for T4 acral melanoma. Meanwhile, five patients whose melanomas were resected with sufficient margins both from nodules and macules showed relatively good survival. A meta-analysis in 2016 [21] that integrated six randomized controlled trials [5,8,9,10,11,12] warns about the potential risk of narrow margin excision for melanoma, providing evidence that 1–2-cm margins may lead to poorer outcomes than 3–5-cm margins. We cannot currently judge whether removal of all local micrometastases in the vicinity of the primary tumor achieved by wide resection led to the better survival outcomes of the five patients because the number of patients is too small. After all, we still do not have enough evidence that warrants narrow-margin excision, and margins following current guidelines would be preferred for curative purpose. In cases with wide in situ macules, optimal surgical margins from nodules and macules may be decided for individual patient. A clinical trial comparing narrow margins (1 cm vs. 2 cm) is ongoing (NCT01457157) and the results of this trial will provide further insights into appropriate surgical margins.

Besides the bias inherent in a retrospective study and the relatively small sample size, one limitation of this study is that dermatologic surgeons determined the surgical margins according to anatomical constraints or patients’ general conditions and wishes. Thus, the rules of the choice of resection margins were inconsistent. Furthermore, in some cases of the narrow-margin excision, surgeons could be less concerned about margins because they considered that a prognosis was going to be poor. All of these factors may have caused uncontrolled bias.

In conclusion, although we could not find a difference between the narrow-margin excision and recommended-margin excision in this study, we suggest following the current recommendations of guidelines. Our study warrants the prospective collection of data on acral melanoma to better define the prognosis of this infrequent type of melanoma.

## Figures and Tables

**Table 1:** Clinicopathological data of patients with invasive acral melanoma.

Parameter	Number (%)
Age in years	
Range (mean ± SD)	16–89 (67.0 ± 15.7)
Sex	
Male	42 (42.0)
Female	58 (58.0)
Histopathological subtype	
Acral lentiginous	100 (100)
Primary tumor site	
Palm	15 (15.0)
Sole	65 (65.0)
Nail bed	20 (20.0)
Ulceration	
Present	49 (49.0)
Absent	51 (51.0)
T category	
T1	31 (31.0)
T2	15 (15.0)
T3	16 (16.0)
T4	38 (38.0)
American Joint Committee on Cancer stage	
I	42 (42.0)
II	30 (30.0)
III	23 (23.0)
IV	5 (5.0)
Melanoma-specific survival in months	
Range (mean ± SD)	1–175 (54.3 ± 43.9)
Disease-free survival in months	
Range (mean ± SD)	0–175 (48.4 ± 46.3)
Total	100 (100.0)

SD: standard deviation.

**Table 2:** Margin status.

T Category (NCCN Recommendation)	Surgical Margin from Tumor Border	Patients (*n*)	Positive Margin (*n*)
T1	5 mm	16	1 ^a^
(10 mm)	≥10 mm	15	1 ^b^
T2	5 mm	3	0
(10–20 mm)	10 mm	8	1 ^c^
	20 mm	4	0
T3	10 mm	11	0
(20 mm)	15 mm	2	0
	20 mm	3	0
T4	5 mm	7	0
(20 mm)	10 mm	20	0
	15 mm	6	0
	≥20 mm	5	0

^a^ Observation without re-excision because of old age. Survival without recurrence for 19 months; ^b^ Re-excision with additional 5-mm margins. Survival without recurrence for 118 months; ^c^ Observation without re-excision because of old age. Survival without recurrence for 61 months; NCCN: National Comprehensive Cancer Network.

**Table 3:** Treatment outcome after follow-up of 95 patiens with stage I–III diseases.

T Category	Surgical Margin (*n*)	Local Recurrence	In Transit Metastasis	Death Due to Melanoma	Follow-Up (Person-Years)
T1	Narrow (16)	1 (6.3%)	0	2 (12.5%)	72.6
	Recommended (15)	0	0	0	72.3
T2	Narrow (3)	0	0	0	7.3
	Recommended (12)	0	0	1 (8.3%)	68.6
T3	Narrow (12)	0	3 (25.0%)	0	67.3
	Recommended (3)	0	1 (33.1%)	1 (33.3%)	15.1
T4	Narrow (29)	1 (3.4%)	4 (13.8%)	14 (48.3%)	122.4
	Recommended (5)	0	0	1 (20.0%)	19.9

**Table 4:** Comparison between narrow and recommended margins for stage I–III patients.

Parameter	Narrow	Recommended	*p*
Age in years			
Mean ± SD	67.3 ± 15.8	66.3 ± 16.8	0.9220
Breslow thickness (mm)			
Mean ± SD	4.21 ± 2.96	2.03 ± 2.20	0.0013 *
Sex			
Male	25	12	0.5194
Female	35	23	
Primary tumor site			
Palm	9	6	0.9540
Sole	38	22	
Nail bed	13	7	
Ulceration			
Present	31	14	0.2947
Absent	29	21	
American Joint Committee on Cancer stage			
I or II	42	30	0.1351
III	18	5	
Total	60	35	

SD: standard deviation. * Significant values

**Table 5:** Comparison between narrow and sufficient margins from nodules for stage I–III patients.

Parameter	Narrow	Sufficient	*p*
Age in years			
Mean ± SD	65.1 ± 16.6	68.7 ± 15.5	0.2264
Breslow thickness (mm)			
Mean ± SD	3.86 ± 3.11	2.62 ± 2.55	0.0633
Sex			
Male	17	20	0.8336
Female	29	29	
Primary tumor site			
Palm	9	6	0.4120
Sole	26	34	
Nail bed	11	9	
Ulceration			
Present	21	24	0.8379
Absent	25	25	
American Joint Committee on Cancer stage			
I or II	33	39	0.4736
III	13	10	
Total	46	49	

SD: standard deviation.

**Table 6:** Cox multivariate analysis for melanoma-specific survival (MSS) and disease-free survival (DFS).

		MSS			DFS	
Variable	HR	95% CI	*p*	HR	95% CI	*p*
Age †	1.05	1.01–1.10	0.0286 *	1.03	0.99–1.06	0.0892
Sex, male	1.87	0.75–4.65	0.1806	1.73	0.82–3.66	0.1528
Breslow thickness †	1.20	1.02–1.40	0.0226 *	1.19	1.05–1.34	0.0045 *
Surgical margin, narrow	1.83	0.47–7.14	0.3836	1.73	0.60–4.99	0.3092

CI, Confidence interval; HR, hazard ratio; † Continuous variables; * Significant values.

**Table 7:** Cox multivariate analysis on the prognostic impact of narrow-margin excision from nodule.

		MSS			DFS	
Variable	HR	95% CI	*p*	HR	95% CI	*p*
Age †	1.05	1.01–1.10	0.0265 *	1.03	0.99–1.06	0.0797
Sex, male	1.99	0.79–5.03	0.1459	1.84	0.86–3.93	0.1169
Breslow thickness †	1.23	1.06–1.42	0.0045 *	1.22	1.08–1.36	0.0006 *
Margin from nodule, narrow	1.29	0.50–3.33	0.5962	1.23	0.56–2.70	0.6087

CI, Confidence interval; HR, hazard ratio; † Continuous variables; * Significant values.

## References

[1] Ferlay J., Soerjomataram I., Dikshit R., Eser S., Mathers C., Rebelo M., Parkin N.M., Forman D., Bray F. (2014). Cancer incidence and mortality worldwide: Sources, methods and major patterns in GLOBOCAN 2012. Int. J. Cancer.

[2] Kohler B.A., Ward E., McCarthy B.J., Schymura M.J., Ries L.A.G., Eheman C., Jemal A., Anderson R.N., Ajani U.A., Edwards B.K. (2011). Annual Report to the Nation on the Status of Cancer, 1975-2007, Featuring Tumors of the Brain and Other Nervous System. J. Natl. Cancer Inst..

[3] NCCN Guidelines Version 3.2020 Cutaneous Melanoma. https://www.nccn.org/professionals/physician_gls/pdf/cutaneous_melanoma.pdf..

[4] Furue M., Ito T., Wada N., Wada-Ohno M., Kadono T., Uchi H. (2018). Melanoma and Immune Checkpoint Inhibitors. Curr. Oncol. Rep..

[5] Cohn-Cedermark G., Rutqvist L.E., Andersson R., Breivald M., Ingvar C., Johansson H., Jönsson P.E., Krysander L., Lindholm C., Ringborg U. (2000). Long term results of a randomized study by the Swedish Melanoma Study Group on 2-cm versus 5-cm resection margins for patients with cutaneous melanoma with a tumor thickness of 0.8-2.0 mm. Cancer.

[6] Mocellin S., Pasquali S., Nitti D. (2011). The Impact of Surgery on Survival of Patients With Cutaneous Melanoma. Ann. Surg..

[7] Lens M.B., Dawes M., Goodacre T., Bishop J.A.N. (2002). Excision Margins in the Treatment of Primary Cutaneous Melanoma. Arch. Surg..

[8] Cascinelli N. (1998). Margin of resection in the management of primary melanoma. Semin. Surg. Oncol..

[9] Balch C.M., Soong S.-J., Smith T., Ross M.I., Urist M.M., Karakousis C.P., Temple W.J., Mihm M.C., Barnhill R.L., Jewell W.R. (2001). Long-Term Results of a Prospective Surgical Trial Comparing 2 cm vs. 4 cm Excision Margins for 740 Patients With 1?4 mm Melanomas. Ann. Surg. Oncol..

[10] Khayat D., Rixe O., Martin G., Soubrane C., Banzet M., Bazex J.-A., Lauret P., Verola O., Auclerc G., Harper P. (2003). Surgical margins in cutaneous melanoma (2 cm versus 5 cm for lesions measuring less than 2.1-mm thick). Cancer.

[11] Thomas J.M., Newton-Bishop J., A’Hern R., Coombes G., Timmons M., Evans J., Cook M., Theaker J., Fallowfield M., O’Neill T. (2004). Excision Margins in High-Risk Malignant Melanoma. N. Engl. J. Med..

[12] Gillgren P., Drzewiecki K.T., Niin M., Gullestad H.P., Hellborg H., Månsson-Brahme E., Ingvar C., Ringborg U. (2011). 2-cm versus 4-cm surgical excision margins for primary cutaneous melanoma thicker than 2 mm: A randomised, multicentre trial. Lancet.

[13] Curtin J., Fridlyand J., Kageshita T., Patel H.N., Busam K.J., Kutzner H., Cho K.-H., Aiba S., Bröcker E.-B., LeBoit P.E. (2005). Distinct Sets of Genetic Alterations in Melanoma. N. Engl. J. Med..

[14] Rabbie R., Ferguson P., Molina-Aguilar C., Adams D.J., Robles-Espinoza C.D. (2019). Melanoma subtypes: Genomic profiles, prognostic molecular markers and therapeutic possibilities. J. Pathol..

[15] Ito T., Kaku-Ito Y., Murata M., Ichiki T., Kuma Y., Tanaka Y., Ide T., Ohno F., Wada-Ohno M., Yamada Y. (2019). Intra- and Inter-Tumor BRAF Heterogeneity in Acral Melanoma: An Immunohistochemical Analysis. Int. J. Mol. Sci..

[16] Ito T., Kaku-Ito Y., Murata M., Furue K., Shen C.-H., Oda Y., Furue M. (2020). Immunohistochemical BRAF V600E Expression and Intratumor BRAF V600E Heterogeneity in Acral Melanoma: Implication in Melanoma-Specific Survival. J. Clin. Med..

[17] Ito T., Wada-Ohno M., Nagae K., Nakano-Nakamura M., Nakahara T., Hagihara A., Furue M., Uchi H. (2015). Acral lentiginous melanoma: Who benefits from sentinel lymph node biopsy?. J. Am. Acad. Dermatol..

[18] Ito T., Wada M., Nagae K., Nakano-Nakamura M., Nakahara T., Hagihara A., Furue M., Uchi H. (2014). Triple-marker PCR assay of sentinel lymph node as a prognostic factor in melanoma. J. Eur. Acad. Dermatol. Venereol..

[19] Wada-Ohno M., Ito T., Tsuji G., Nakahara T., Hagihara A., Furue M., Uchi H. (2017). Acral lentiginous melanoma versus other melanoma: A single-center analysis in Japan. J. Dermatol..

[20] Bajaj S., Donnelly D., Call M., Johannet P., Moran U., Polsky D., Shapiro R., Berman R., Pavlick A., Weber J. (2020). Melanoma Prognosis: Accuracy of the American Joint Committee on Cancer Staging Manual Eighth Edition. J. Natl. Cancer Inst..

[21] Wheatley K., Wilson J.S., Gaunt P., Marsden J.R. (2016). Surgical excision margins in primary cutaneous melanoma: A meta-analysis and Bayesian probability evaluation. Cancer Treat. Rev..

